# Integral assessment of gas exchange during veno-arterial ECMO: accuracy and precision of a modified Fick principle in a porcine model

**DOI:** 10.1152/ajplung.00045.2022

**Published:** 2022-12-13

**Authors:** David C. Berger, Lena Zwicker, Kay Nettelbeck, Daniela Casoni, Paul Phillipp Heinisch, Hansjörg Jenni, Matthias Haenggi, Luciano Gattinoni, Kaspar F. Bachmann

**Affiliations:** ^1^Department of Intensive Care Medicine, Inselspital, Bern University Hospital, University of Bern, Bern, Switzerland; ^2^Experimental Surgery Facility (ESF), Department for BioMedical Research, Faculty of Medicine, University of Bern, Bern, Switzerland; ^3^Department of Congenital and Pediatric Heart Surgery, German Heart Center Munich, Technische Universität München, Munich, Germany; ^4^Department of Anesthesiology & Pain Medicine, Inselspital, Bern University Hospital, University of Bern, Bern, Switzerland; ^5^Department of Anesthesiology, Medical University of Göttingen, University Medical Center Göttingen, Göttingen, Germany

**Keywords:** cardiac output, extracorporeal membrane oxygenation, Fick principle, gas exchange, ventilation/perfusion mismatch

## Abstract

Assessment of native cardiac output during extracorporeal circulation is challenging. We assessed a modified Fick principle under conditions such as dead space and shunt in 13 anesthetized swine undergoing centrally cannulated veno-arterial extracorporeal membrane oxygenation (V-A ECMO, 308 measurement periods) therapy. We assumed that the ratio of carbon dioxide elimination (V̇co_2_) or oxygen uptake (V̇o_2_) between the membrane and native lung corresponds to the ratio of respective blood flows. Unequal ventilation/perfusion (V̇/Q̇) ratios were corrected towards unity. Pulmonary blood flow was calculated and compared to an ultrasonic flow probe on the pulmonary artery with a bias of 99 mL/min (limits of agreement −542 to 741 mL/min) with blood content V̇o_2_ and no-shunt, no-dead space conditions, which showed good trending ability (least significant change from 82 to 129 mL). Shunt conditions led to underestimation of native pulmonary blood flow (bias −395, limits of agreement −1,290 to 500 mL/min). Bias and trending further depended on the gas (O_2_, CO_2_) and measurement approach (blood content vs. gas phase). Measurements in the gas phase increased the bias (253 [LoA −1,357 to 1,863 mL/min] for expired V̇o_2_ bias 482 [LoA −760 to 1,724 mL/min] for expired V̇co_2_) and could be improved by correction of V̇/Q̇ inequalities. Our results show that common assumptions of the Fick principle in two competing circulations give results with adequate accuracy and may offer a clinically applicable tool. Precision depends on specific conditions. This highlights the complexity of gas exchange in membrane lungs and may further deepen the understanding of V-A ECMO.

## Graphical abstract

**Figure:**
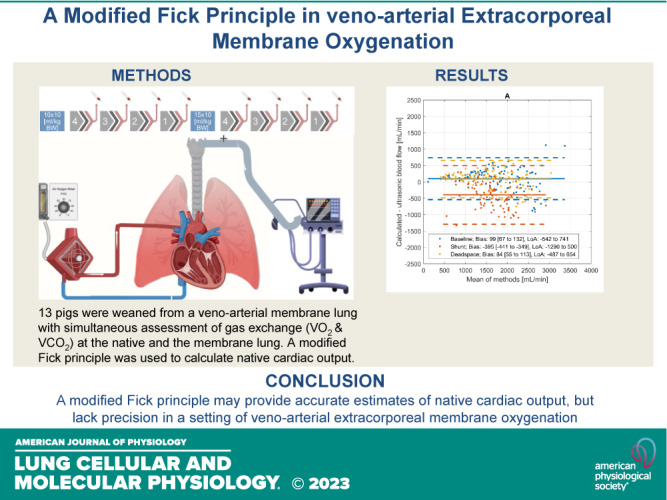

## INTRODUCTION

Veno-arterial extracorporeal membrane oxygenation (V-A ECMO) has been increasingly used as a rescue strategy in intensive care medicine for severe cardiopulmonary failure in the last decade (1). Particularly, the use of extracorporeal life support in the setting of cardiopulmonary resuscitation and postcardiotomy cardiogenic shock are established concepts (2, 3). Mortality and morbidity with this treatment modality remain excessively high (up to 50%) (4, 5). Despite their paramount importance in guiding therapy, standard hemodynamic monitoring methods of the patient’s conditions are influenced by the altered physiology on ECMO. It is increasingly recognized that standard methods for continuous cardiac output monitoring like transpulmonary thermodilution (6) or the pulmonary artery catheter lack validation (7, 8) and may therefore not serve their intended purpose during ECMO. Our study group has recently demonstrated that classic approaches of transcardiac thermodilution are not valid without conceptual adaptations in the setting of V-A ECMO (9). Echocardiography as a recommended tool remains noncontinuous and user-dependent (8). The precise measurement and monitoring of native cardiac output was identified as an urgent clinical need (10).

Assessment of alveolar gas exchange is a traditional physiological method for measuring cardiac output, with various techniques (11). Expiratory gas measurements are readily available in intensive care and operating theaters. These may offer a new, standardized, noninvasive and continuous monitoring technique for cardiac output measurement in the context of ECMO. We have recently applied such methodology using a modified Fick principle and carbon dioxide measurements in a proof-of-concept animal model of V-A ECMO with specific adaptations regarding the extracorporeal component under healthy conditions (12). In an in vitro model, we showed that this modified Fick approach based on oxygen blood content measurements allows the calculation of cardiac output with a very small bias (7).

To assess measurements of native cardiac output through gas exchange parameters during V-A ECMO further, this current study addresses this research question in a large sample of animals in a well-controlled experimental setting and defines the critical steps for the development of a method usable at the bedside. First, we determined the performance of the modified Fick approach based on blood content measurements, including baseline, dead space, and shunt conditions. This assessed the performance under common pathophysiological states affecting the gas exchange. Second, to enable continuous measurements of cardiac output at the bedside, we assessed the performance of the modified Fick principle in the gas phase. Therefore, we compared calculations for blood content and gas content for respiratory gas measurements and the respective effects on the performance of the cardiac output calculations. This stepwise approach enabled us to describe the inherent physiological limitations of our modified Fick technique, the limitations in measurement techniques, and the resulting relationship between gaseous measurements of oxygen and carbon dioxide and measurements in the blood phase. Finally, our extensive data set has enabled the description of in vivo extracorporeal gas exchange, providing real-life insights into gas exchange during V-A ECMO therapy that is comparable to mathematical models.

## METHODS

The Commission of Animal Experimentation of Canton Bern, Switzerland, approved this study (BE111/18) in compliance with Swiss national guidelines and the *Guide for the Care and Use of Laboratory Animals* (National Academy of Sciences, 1996). This report follows the applicable ARRIVE (animal research: reporting of in vivo experiments) guidelines. The data that support the findings of this study are available from the corresponding author on reasonable request. Data from this study on an adapted thermodilution technique have been published separately (9).

### Anesthesia and Surgery

**Figure 1.**
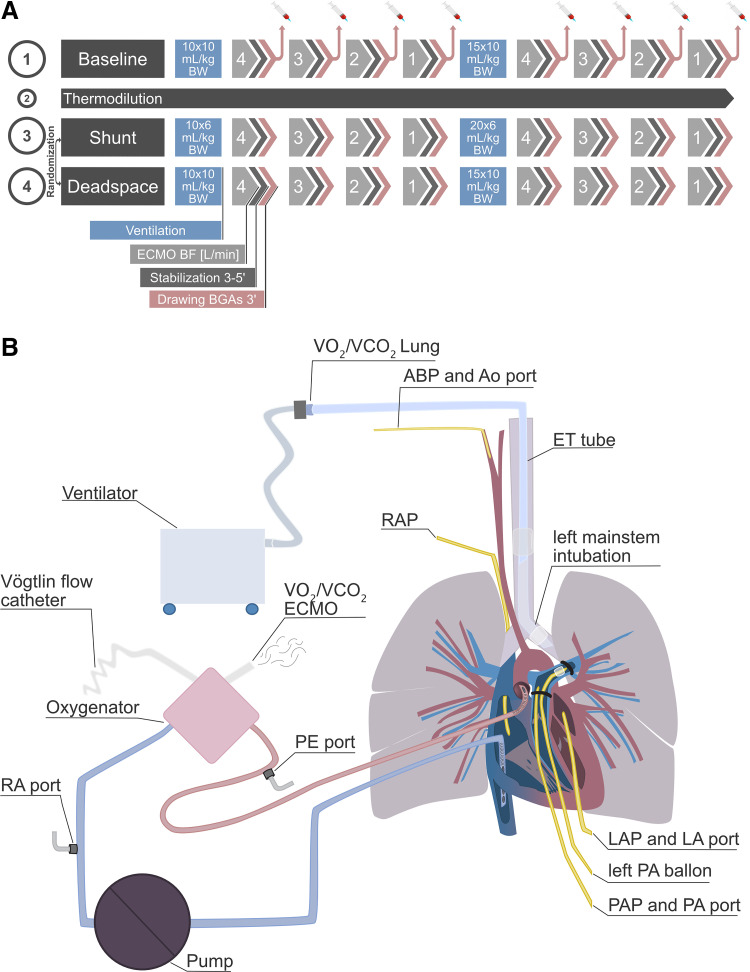
*A*: experimental setup. *B*: experimental conditions. ABP, arterial/systemic blood pressure; AO, aorta; BF, blood flow; BGA, blood gas analysis; BW, body weight; ECMO, extracorporeal membrane oxygenation; LA, left atrium; LAP, left atrial pressure; PA, pulmonary artery; PAP, pulmonary artery pressure; RA, right atrium; RAP, right atrial pressure.

After premedication with ketamine, 16 healthy pigs (“Schweizer Edelschwein,” *Sus scrofa*, 45.5 kg [42–47 kg], 10 females) were anesthetized and ventilated (Hamilton C6, volume control mode, positive end-expiratory pressure 5 cm H_2_O, $FI_{O_{2}}$ 0.6). The animals underwent central V-A ECMO cannulation after sternotomy and heparinization (Fig. 1*A*). The details of animal care and the anesthesiologic and surgical management of this experiment have been previously published (9).

The right atrium and ascending aorta were cannulated (29 Fr 3-stage venous cannula MC2X and 18 Fr elongated one-piece arterial cannula, Medtronic, Minneapolis, MN) and connected to an ECMO circuit (Stöckert SCPC console, LivaNova, London, England and Revolution centrifugal blood pump, LivaNova, London, England with CAPIOX FX15 oxygenator, Terumo, NJ). Transit time ultrasonic flow probes (Transonic PAU series, Ithaca, NY, size 20/18 mm and 8 mm) were mounted around the pulmonary artery main trunk and the left pulmonary artery and the ECMO return cannula (Transonic ME9PXL1507, Ithaca, NY). A Fogarty balloon catheter was placed in the left pulmonary artery for intermittent partial occlusion. A 1-lumen central venous line was placed in the left atrium for pressure measurement. Another 1-lumen catheter line was inserted surgically in the right ventricle. Temperature was kept at 37.0°C using a temperature control system (HCV, Type 20–602, Jostra Fumedica, Muri, Switzerland).

Sweep gas was blended from air and oxygen with two respective mass flow controllers (Vögtlin RED-Y, Basel-Land, Switzerland) to achieve a constant inlet oxygen concentration ($Fd_{O_{2}}$) of 0.6. Sweep gas flow was always set to match blood flow at the ECMO [resulting in a ventilation/perfusion (V̇/Q̇_ECMO_) ratio of 1] with reductions in sweep gas flow only during stabilization periods between experimental maneuvers to achieve a pH of 7.4–7.5 (13).

### Experimental Protocol

After surgery, instrumentations were controlled using fluoroscopy. During a stabilization period of 60 min, all devices were calibrated and measurements initiated. The experimental protocol (Fig. 1*B*) consisted of four phases: baseline, thermodilution, shunt, and dead space in randomized order. The thermodilution phase has been analyzed and published separately (9) and is not discussed here. Dead space was created through intermittent inflation of the Fogarty balloon catheter in the left pulmonary artery, and shunt was achieved by selective intubation of the left main bronchus. The flow probe on the left pulmonary artery confirmed the effect of these maneuvers. The baseline, shunt, and dead space phases consisted of four ECMO blood flow reductions ranging from 4 L/min to 1 L/min (1-L/min steps). Sweep gas flow was set to match blood flow (V̇/Q̇_ECMO_ =1). After each such flow reduction, the animal was allowed to stabilize for 5 min before a measurement period of 3 min (resulting in four measurement steps per phase). In that period, five blood gas samples were drawn and measurements of gas exchange at the ventilator and the ECMO were recorded (see *Blood Gas Measurements* and *Assessment of Gas Exchange*). Each phase was repeated twice (i.e., baseline 1, baseline 2, shunt 1, shunt 2, dead space 1, dead space 2) with various ventilator settings to vary the V̇/Q̇ ratio at the lung. For baseline 1 and 2 as well as dead space 1 and 2, the ventilator was set to 10 × 10 mL/kg body wt (kilogram of body weight) and 15 × 10 mL/kg body wt, respectively. For shunt 1 and 2, the ventilator was set to 10 × 6 mL/kg body wt and 20 × 6 mL/kg body wt, respectively. The ratio of inspiratory to expiratory time was constant at 1:1.6.

At the end of the experiments, the animals were euthanized in deep anesthesia with an injection of 1 mmol/kg potassium chloride under the monitoring of the electrocardiogram and electroencephalogram to ensure asystole and brain death.

### Blood Gas Measurements

Blood gas samples were collected simultaneously by two qualified intensive care nurses at five ports: at the pulmonary artery (PA), left atrium (LA), ECMO inlet/right atrium (RA), postoxygenator (PE), and aorta (AO; Fig. 1*A*). The samples were sealed airtight and immediately cooled for analysis within minutes. Depending on the availability on a respective day, either the cobas b123 POC (Roche Diagnostics, Basel, Switzerland) or ABL90 FLEX (Radiometer Medical, Kopenhagen, Denmark) blood gas analyzer was used for the following measurements: partial pressure of carbon dioxide (Pco_2_), partial pressure of oxygen (Po_2_), hematocrit (Hct), hemoglobin (Hb), oxygen saturation (So_2_), pH, and standard bicarbonate. Both devices have an integrated CO-oxymeter for the measurement of oxygen saturation with coefficients for human hemoglobin. Since significant interspecies differences exist for these coefficients, all So_2_ values were corrected for pigs using Serianni’s approach (14).

### Assessment of Gas Exchange

Breath-by-breath CO_2_ measurements and pulmonary elimination of CO_2_ (V̇co_2Lung_) were performed using a Capnostat 5 capnograph (Hamilton Medical, Bonaduz, Switzerland) at the endotracheal tube. For O_2_ uptake in the lung (V̇o_2Lung_), the delay in the side-stream O_2_ signal was time matched to the mainstream CO_2_ signal and then integrated with gas flow at the tracheal tube (15). At the ECMO circuit, a side-stream module for indirect calorimetry (E-COVX, General Electric, Baden, Switzerland) for continuous measurement of membrane lung exhaust gas was attached. In the gas phase, V̇co_2_ was measured by integrating tidal gas flow with the mainstream capnography and sweep flow with the exhaust capnography signal (15, 16). Haldane’s transformation was used to calculate postoxygenator gas flow as follows (assuming a nitrogen concentration of 0.79 at the air-fed flow controller) (17):

(*1*) $$N_{2}\;inflow={Air\;Flow}_{Inlet}\times Nitrogen\;Concentration$$

(*2*) $${Total\;Gas\;Flow}_{Outlet}=\frac{N_{2}inflow}{1-F_{PE}O_{2}-F_{PE}CO_{2}}$$

Calibrations were performed on 7 of 16 experimental days using predefined mixtures of CO_2_/N_2_/O_2_.

### Data Acquisition

Measurements included sweep gas and blood flow at the ECMO, blood flow through the lungs, airway pressures, tidal volumes and flow, pulmonary end-tidal oxygen and carbon dioxide tension ($\mathrm{PET}O_{2}$, $\mathrm{PET}{\mathrm{CO}}_{2}$), pressures from the carotid artery, right atrium, left atrium, and pulmonary artery, body temperature, ECMO exhaust oxygen and carbon dioxide tensions, and continuous oximetric mixed venous saturation (calibrated every 3 h). Three-minute measurement intervals were used.

All data output from temperature probes, pressure transducers, and ultrasonic blood flow probes were recorded at 100 Hz in LabVIEW (National Instruments Corp., Austin, TX) and Soleasy (Alea Solutions, Zürich, Switzerland). Data from the respirator were recorded at 50 Hz using a dedicated recording device (Memory Box, Hamilton Medical, Bonaduz, Switzerland).

### Formulas and Calculations

Mass balance demands that in a steady state, total carbon dioxide production (V̇co_2_) and total oxygen consumption (V̇o_2_) have to match the elimination of V̇co_2_ at the lung plus ECMO and the oxygen uptake at the lung plus ECMO, respectively. Using the Fick principle, *Eq. 3A* can be deducted (12), where Δc_V-AO_ refers to the veno-arterial content difference, Δc_V-LA_ refers to the content difference over the pulmonary circulation, and Δc_V-PE_ refers to the content difference over the ECMO circuit. Q̇ is the blood flow. In theory, CO_2_ can be substituted for any other gas that is in equilibrium (*Eq. 3B*):

(*3A*) $${\dot{Q}}_{total}\times \;{\Delta c}_{\bar{v}-AO}{CO}_{2}\;=\;{\dot{Q}}_{Lung}\times \;{\Delta c}_{\bar{v}-LA}{CO}_{2}+\;{\dot{Q}}_{ECMO}\times \;{\Delta c}_{\bar{v}-PE}{CO}_{2}\;$$

(3*B*) $${\dot{Q}}_{total}\times \;{\Delta c}_{\bar{v}\text{-}AO}O_{2}\;=\;{\dot{Q}}_{Lung}\times \;{\Delta c}_{\bar{v}-LA}O_{2}+\;{\dot{Q}}_{ECMO}\times \;{\Delta c}_{\bar{v}-PE}O_{2}\;$$

Rearrangement of *Eqs. 3A* and *3B* proposes a proportional relationship of oxygen consumption/carbon dioxide elimination and respective blood flows under the assumption that ${\dot{Q}}_{total}={\dot{Q}}_{Lung}+\;{\dot{Q}}_{ECMO}$:

(*4*) $${\dot{Q}}_{Lung}={\dot{Q}}_{ECMO}\times \frac{\left(\Delta_{\bar{v}-PE}{CO}_{2}-\;\Delta_{\bar{v}-AO}{CO}_{2}\right)}{\left(\Delta_{\bar{v}-AO}{CO}_{2}-\;\Delta_{\bar{v}-LA}{CO}_{2}\right)}\;$$

V̇co_2_ is the product of the differences in CO_2_ content times the blood flow and thus *Eq. 4* can be simplified using the following assumptions: ${\dot{V}{co}_{2}}_{ECMO}$ is proportional to △_V-PE_CO_2_, ${\dot{V}{co}_{2}}_{Lung}$ is proportional to △_V-LA_CO_2_, ${\dot{V}{co}_{2}}_{Total}$ is proportional to △_V-AO_CO_2_, and the total V̇co_2_ is the sum of V̇co_2_ at the ECMO and the lung. As production and elimination are mathematical opposites, we use absolute values (12).

(*5*) $${\Delta \dot{Q}}_{Lung}={\dot{Q}}_{ECMO}\times \frac{\left|{\dot{V}{CO}_{2}}_{Lung\;}\right|}{{|\dot{V}{CO}_{2}}_{ECMO}|}$$

(*6*) $${\Delta \dot{Q}}_{Lung}={\dot{Q}}_{ECMO}\times \frac{\left|{\dot{V}O_{2}}_{Lung}\right|}{\left|{\dot{V}O_{2}}_{ECMO}\right|}$$

The original Fick principle leads to the assumption that *Eqs. 5** and **6* also hold true for oxygenation and O_2_ consumption (V̇o_2_, *Eq. 4*). The full derivation of these equations can be found in Bachmann et al. (12).

The method of Dash and Bassingwaithe was used to calculate blood CO_2_ content (cCO_2_) (18). Oxygen content (cO_2_) was calculated using the standard formula (*Eq. 7*):

(*7*) $${cO}_{2}=1.36\times Hb\times \frac{{So}_{2}}{100}+0.003\times {Po}_{2}\;$$

In the blood phase, V̇co_2_ and V̇o_2_ were calculated as the difference between arterial and venous content times measured blood flow.

V̇co_2_ is highly dependent on the V̇/Q̇̇ ratio and is thus dependent not only on blood flow but also on ventilation (19). This interferes with the precision of blood flow calculations, as the constructed mass balance equation is only applicable if the inflow and outflow gas content of both the ECMO and the lung are equal. To correct for this, an empirical approach for normalizing V̇co_2_ at the lung was applied (12).

(*8*) $$f\left(\dot{V},\dot{Q}\right)=\;\frac{\dot{Q}\times \left(\frac{\dot{V}}{\dot{Q}}+c\right)}{\dot{V}\times \left(1+c\right)}=\frac{\left(\frac{\dot{V}}{\dot{Q}}+c\right)}{\left(1+c\right)}\times \frac{1}{\frac{\dot{V}}{\dot{Q}}}$$

The constant c was calculated from a venous blood gas sample [$c=\sigma_{CO2}\times R\times T\times \left(1+K_{c}\right)]$ as a function of temperature T, pH (K_c_), CO_2_ solubility (σ_CO2_), and the gas constant R (20). In brief, this normalization allows the calculation of V̇co_2_ that is dependent on blood flow only and independent of ventilation; V̇co_2_ is therefore normalized toward a V̇/Q̇ ratio of 1. To estimate V̇/Q̇ at the lung, we used a previously described mass balance equation (21):

(*9*) $$\frac{\dot{V}}{\dot{Q}}=\frac{{FI}_{O_{2}}-{FE}_{O_{2}}}{c_{v}O_{2}-c_{a}O_{2}}$$

Blood gas calculations were performed using either V̇o_2_ calculated as the product of blood gas O_2_ content difference times blood flow (V̇o_2 Blood_), V̇o_2_ measured in the gas phase (V̇o_2 Gas_), V̇co_2_ measured in the gas phase (V̇co_2 Gas_), normalized V̇co_2_ in the gas phase (V̇co_2 Gas Norm_), or V̇co_2_ in the blood phase (V̇co_2 Blood_). Calculated blood flows were compared to measured blood flows for each experimental phase separately.

Arterial and venous oxygen content (c_a_O_2_, c_v_O_2_) were calculated from the blood gas samples. Normalization at the ECMO was not applied, as V̇/Q̇ was specifically chosen to be 1.

### Statistical Analysis

Analyses were performed using MATLAB R2021a (MathWorks, Natick, MA) with an extension for Bland–Altman plots under creative commons license (22). Data are presented as means with standard deviations or as medians with interquartile ranges where appropriate. Method agreement was tested with linear regression (least squares method) and Bland–Altman analysis (23, 24). A two-tailed *P* value of < 0.05 was considered statistically significant. The least significant change of a method was calculated according to standard methods (25). The relationship between V̇co_2_ and V̇o_2_ and the underlying parameters (blood gas content and blood flow) were assessed using linear mixed-effect models. Outliers were removed according to the following rules: calculated blood flow > 10 L/min or negative calculated pulmonary blood flows. The sample size was calculated from data from pilot animals, which showed an increase in pulmonary blood flow of 1 L/min for each 1 L/min reduction in ECMO flow. To detect these changes appropriately with a precision error of < 30%, we calculated a sample size of 10 animals. To compensate for an expected dropout rate of 20% and to establish the experimental setup in four pilot animals, the experiment was performed in 16 animals.

## RESULTS

### Summary of Experiments

After exclusion of one dead space maneuver (animal 5, due to technical recording problems), data from 13 animals with 308 measurement periods were analyzed, resulting in a total of 1,540 blood gas samples (7 animals with Radiometer, 9 animals with the cobas). ECMO flow was within the targets set by the protocol. Blood flow through the lung increased from median values of 833–1,216 (ECMO flow 4 L/min) to median values of 2,257–2,541 mL/min with ECMO flow reductions (1 L/min), depending on the experimental condition. With these flow changes, V̇o_2_ and V̇co_2_ changed accordingly with increases in V̇o_2_ and V̇co_2_ at the lung and decreases in V̇o_2_ and V̇co_2_ at the ECMO (Supplemental Table S1). Total V̇o_2_ was ∼200 mL/min, which corresponds to physiological values for pigs (13). Total V̇co_2_ at the initial ECMO flow of 4 L/min was around 250 mL/min, resulting in a respiratory exchange ratio of ∼0.8. Total O_2_ consumption and CO_2_ removal did not remain constant throughout ECMO flow changes. Although V̇o_2 Total_ measured in the blood phase remained mostly unchanged, there was a decrease in CO_2_ removal (V̇co_2 Total_) through the decrease in ECMO blood flow (Supplemental Fig. S1).

### Cardiac Output Estimates Based on Oxygen Content

**Figure 2.**
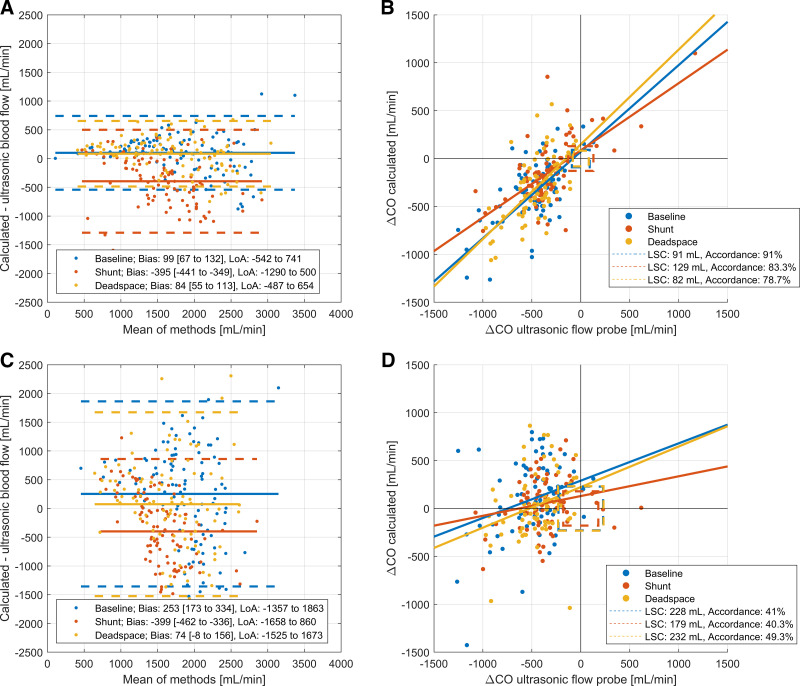
Cardiac output calculations based on oxygen content. *A*: Bland–Altman plot for calculated cardiac output using blood measurements of V̇o_2Blood._ Percentage errors: Baseline 36.4%. Shunt 51.0%. Dead space 35.1%. Regression equations are given in the online supplement. *B*: trending abilities for V̇o_2Blood._ Regression equations are given in the online supplement. *C*: Bland–Altman plot for calculated cardiac output using blood measurements of V̇o_2Gas._ Percentage errors: Baseline 91.4%. Shunt 71.3%. Dead space 98.8%. Regression equations are given in the online supplement. *D*: trending abilities for V̇o_2 Gas._ Regression equations are given in the online supplement.

The first step was to test the modified Fick approach with oxygen content measurements in blood (V̇o_2 Blood,_ Fig. 2*A*; see also Supplemental Table S2). The resulting cardiac output showed low bias with narrow limits of agreement for baseline (Bias 99 mL, LoA −542 to 741 mL) and dead space conditions (bias 84 mL, LoA −487 to 654 mL). Shunt conditions lead to considerable loss in precision and accuracy (bias −395 mL, LoA −1,290 to 500 mL). The trending ability of these cardiac output calculations (i.e., the accordance between calculated changes and measured changes in blood flow) was high (least significant changes from 82 to 129 mL, Fig. 2*B*).

Since continuous measurements are not possible in the blood, measurements in the gas phase would be of interest. The relationship between V̇o_2_ in expired or exhaust air and V̇o_2Blood_ is accurate but lacks precision (ECMO data: bias 10 mL/min with limits of agreement of −50 to 70 mL/min; lung data: bias 9 mL/min with limits of agreement of −43 to 61 mL/min; Supplemental Fig. S2, *A* and *B*).

Cardiac output estimates based on oxygen uptake measurements in the gas phase (V̇o_2Gas,_ Fig. 2*C*) provide acceptable accuracy, but low precision (Baseline: bias 253 mL, LoA −1,357 to 1,863 mL; Shunt: Bias −399 mL, LoA −1,658 to 860 mL; Dead space: bias 74 mL, LoA −1,575 to 1,673 mL) and low accordance rates for trending (40 to 49%, Fig. 2*D*).

### Cardiac Output Measurements Based on Carbon Dioxide Contents

V̇co_2 Blood_ showed considerable deviation from V̇co_2gas_ (bias −100 mL/min, LoA −222 to 21 mL/min for ECMO data and bias −10 mL/min, LoA 97 to 77 mL/min for lung data, Supplemental Fig. S2, *C* and *D*). We suspect that this lack of agreement lays in the blood content model used (18). Since at the bedside, the gas measurement would allow continuous information, and if blood measurements were available, oxygen content may be used, we omit further use of blood flow calculations using blood measurements of CO_2_.

**Figure 3.**
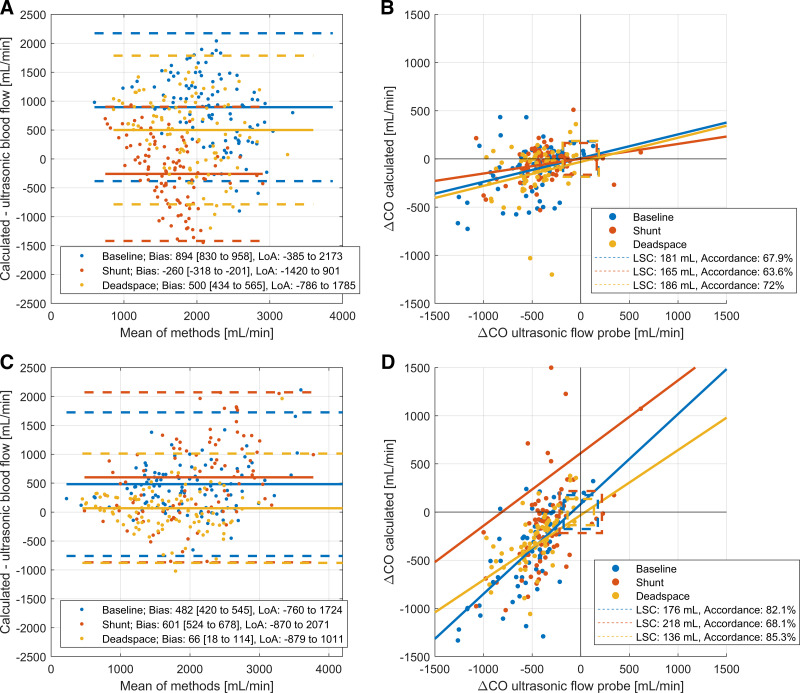
Cardiac output calculations based on carbon dioxide content. *A*: Bland–Altman plot for calculated cardiac output using measurements of V̇co_2 Gas._ Percentage errors: Baseline 72.6%. Shunt 70.5%. Dead space 79.2%. *B*: trending abilities for V̇co_2 Gas_. Regression equations are given in the online supplement. *C*: Bland–Altman plot for calculated cardiac output using measurements of V̇co_2 Gas Norm_. Percentage errors: Baseline 70.5%. Shunt 58.2%. Dead space 83.2%. *D*: trending abilities for V̇co_2 Gas Norm._ Regression equations are given in the online supplement.

Cardiac output estimates resulting from V̇co_2gas_ measurements at the lung showed considerable bias with wide limits of agreement for baseline (Bias 894 mL, LoA −385 to 2,173 mL) and dead space (bias 500 mL, LoA −786 to 1,758 mL) and Shunt (bias −260 mL, LoA −1,420 to 901 mL). The trending ability of these cardiac output calculations was moderate (least significant changes from 165 to 1,186 mL, Fig. 3, *A* and *B*; see also Supplemental Table S3).

Empirical normalization for the V̇co_2Lung_ (12) decreased the bias and narrowed the limits of agreement for baseline (Bias 482 mL, LoA −760 to 1,724 mL), dead space conditions (bias 66 mL, LoA −879 to 1,011 mL), and shunt conditions (bias 601 mL, LoA −870 to 2,071 mL). Of note, V̇co_2 Gas Norm_ showed increased accordance and trending ability compared to calculations through V̇co_2 Gas_ and V̇o_2Gas_ (Fig. 3, *C* and *D*).

### Isolating the Limiting Factors

**Figure 4.**
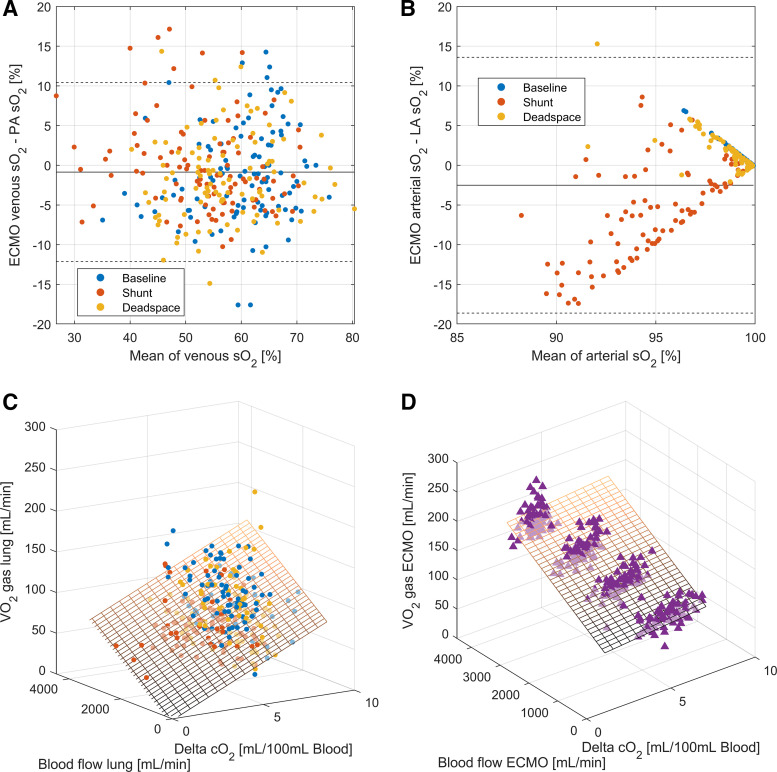
Influencing factors on the cardiac output calculations based on oxygen content. Inflow (venous ECMO drainage and pulmonary artery) and outflow (arterial ECMO limb and left atrium) blood gas content for venous oxygen saturation (So_2_; bias −0.9% [−1.2 to −0.5%], limits of agreement −11.2 to 11.4%; *A*) and arterial So_2_ (bias –2.5% [−3.0 to −2.0%], limits of agreement −18.6 to 13.6%; *B*). Linear mixed-effect models assessing the relationship between difference in blood gas content, blood flow, and gaseous measurements of gas exchange for V̇o_2Lung_ (*C*) and V̇o_2ECMO_ (*D*). The planes represent the model estimates. Model parameters are given in the online supplement. ECMO, extracorporeal membrane oxygenation; LA, left atrium; PA, pulmonary artery.

**Figure 5.**
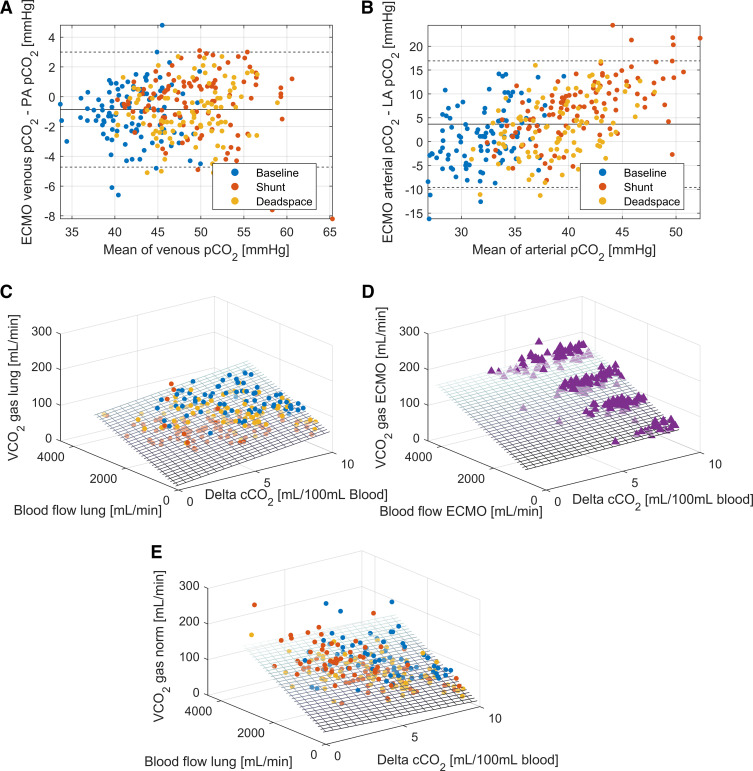
Influencing factors on the cardiac output calculations based on carbon dioxide content. Inflow (venous ECMO drainage and pulmonary artery) and outflow (arterial ECMO limb and left atrium) blood gas content for venous partial pressure of carbon dioxide (Pco_2_; bias −0.86 mmHg, limits of agreement –4.72 to 3.00 mmHg; *A*) and arterial Pco_2_ (bias 3.66 mmHg [3.28 to 4.05 mmHg], limits of agreement −9.59 to 16.92 mmHg; *B*). Linear mixed-effect models assessing the relationship between difference in blood gas content, blood flow, and gaseous measurements for V̇co_2Lung_, (*C*) V̇co_2ECMO_, (*D*), and V̇co_2Norm Lung_ (*E*). The planes represent the model estimates. Model parameters are given in the online supplement. ECMO, extracorporeal membrane oxygenation; LA, left atrium; Norm, normalized; PA, pulmonary artery.

The inflow (venous) and outflow (arterial) conditions between ECMO and lung were inhomogeneous. Venous oxygen saturations and Pco_2_ values differed substantially between ECMO drainage (right atrium) and the pulmonary artery (Fig. 4, *A* and *B* and Fig. 5, *A* and *B*). The difference in outflow saturations is relevant only for the shunt condition, where, as consequence of the experimental condition, saturations below 100% for left atrial blood occurred (Fig. 4*B*). The differences in arterial Pco_2_ values are a direct result of the V̇/Q̇ ratios being unequal to 1 at the lung (Fig. 5*B*).

Using linear-mixed effect models, the factors influencing these wide limits of agreement and bias were assessed. The models show a significant relationship between gaseous measurements and blood content difference and blood flow. The relationship differs depending on the measurement technique. V̇co_2 Gas Norm_ and V̇co_2 Gas ECMO_ are predominantly blood-flow dependent (Fig. 5, *D* and *E*), whereas the other modalities are both influenced by blood flow and blood content differences (Fig. 4, *C* and *D* and Fig. 5*C*). All models were significant, with *r*^2^ from 0.58 to 0.59 for lung gas exchange models and 0.69 to 0.95 for models of gas exchange at the ECMO (equation data in Supplemental Table S4). The calculated V̇/Q̇ ratio used for the V̇co_2_ normalization shows a median value of 1.26 for baseline conditions, 0.58 for shunt conditions, and 1.43 for dead space conditions (Supplemental Fig. S3).

## DISCUSSION

In this study, we could show that a modified Fick principle estimates native cardiac output with high accuracy but considerable lack in precision in the setting of VA-ECMO. Shunt creation led to systematic underestimation of true blood flow, whereas dead space creation did not change the accuracy of the method. Accuracy and precision of the cardiac output estimates depend on the measurement technique (blood vs. gas phase) and the gas chosen (oxygen vs. carbon dioxide) and will be further elaborated below.

As a major limitation of the experimental setup, we used healthy animals in our model. This allowed standardized investigations and controlled induction of shunt and dead space, but may not resemble clinical situations of complex disturbances in gas exchange by either shock or lung failure. Further limitations are the use of a side-stream capnograph for oxygen measurements and the lack of appropriate blood content models for pigs, particularly carbon dioxide. We could also not verify our estimates of the V̇/Q̇̇ ratio at the lung, which we needed for the normalization procedure, with an independent technique like MIGET or impedance tomography.

Cardiac output measurements are notoriously imprecise. Limits of agreement of ±30% are considered the limit for the acceptance of a new measurement method for clinical use (26), but even larger bands up to 45% are discussed. For V̇o_2Blood_ (i.e., a classic Fick approach), we reached clinically acceptable accuracy and precision, with acceptable trending ability (25). For other modalities of gas exchange, particularly measurements in the gas phase, the results are limited for clinical application by their lack of precision. Although gaseous measurements are noninvasive and could be easily implemented in a clinical setting, our results show their inherent limitations. The results are highly dependent on the measurement technique for exhaust and exhaled gases. The normalization procedure, although dependent on an estimation of V̇/Q̇ at the lung, which we could not verify independently, improves the accuracy and precision substantially.

We identify three substantial contributors to the imprecision in blood flow calculations based on our modified Fick principle: First, the measurement techniques (capnography and derived oxygen consumption) have inherent inaccuracies. The capnostat 5 device has a measurement accuracy of ± 2 mmHg below 41 mmHg and ± 5% of the reading in the range of 41 to 71 mmHg. The manufacturer of the E-COVX side-stream module reports an accuracy of ± (0.2 vol % + 2% of the reading) for carbon dioxide and ± (1 vol% + 2% of the reading) for oxygen. In addition, synchronization problems of expiratory tidal flow and side-stream measurements of oxygen may have occurred. Both may be overcome in the future by more precise measurement probes and the use of volumetric capno- or oxygraphs.

Furthermore, gas content calculations of carbon dioxide are insufficient and only human models exist (27). The error in blood content resulting from the transfer between blood and gas measurements is described in Supplemental Fig. S2. Linear mixed-effect models (Figs. 4 and 5) reveal a significant relationship between the relevant factors determining V̇o_2_/ V̇co_2_. Gaseous measurements at the ECMO outlet are more accurate, because there is constant flow and the applied Haldane transformation allows precise calculation of gas flow at the ECMO outlet with oxygen concentrations below 0.6.

The ability of an ECMO system to remove carbon dioxide at high V/Q ratios leads to the clinical commonplace that carbon dioxide elimination is completely independent of blood flow, which is not the case. V̇/Q̇̇ ratios that are unequal to 1 will influence the amount of carbon dioxide removed from the blood and thus interfere with the accuracy of the presented method. Similarly, low V̇/Q̇ ratios at the lung lead to low oxygen saturations (Fig. 4*B*). The normalization procedure can empirically correct for this but is dependent on the accuracy of the V̇/Q̇ estimation at the lung, which we could not verify independently. Of note is the corrected bias presented in Fig. 3*C* for the shunt condition, which shows the effect of normalization. Although the bias for shunt in general is negative because of an expected underestimation resulting from blood flow not participating in gas exchange, the normalization procedure can correct not only for high V̇/Q̇ (> 1) but also for this low V̇/Q̇ distribution. The effect of the normalization procedure is visualized through the linear mixed-effects model in Fig. 5*E*, where V̇co_2_ becomes dependent only on blood flow and is independent of differences in gas content, which allows for better precision in blood flow calculations.

The proposed mass balance equations (*Eqs. 3A and 3B*) are true only if both circuits are participating in the gas exchange of the entire body, resulting in equal inflow conditions, and if the amount of gas exchanged is the same, resulting in equal outflow conditions. As shown in Figs. 4 and 5, this is the case for neither oxygen nor carbon dioxide in our experimental setup. Although clinicians are familiar with the differences in the outflow content and recognize it at the bedside as differential hypoxia or the Harlequine phenomenon (28, 29), the content differences on the venous side are less known. These may have a considerable impact on cannulation choice and effectiveness (30) and may even lead to wrong clinical conclusions when inflow saturation are considered as a surrogate for true mixed venous blood (i.e., blood from the pulmonary artery), as it is recommended practice (8). It may be of further note that the usual calculations for gas exchange calculations on ECMO (31, 32) usually do not consider differences in inflow (“mixed”) venous saturations or carbon dioxide concentrations.

Third, we have previously used differences in V̇co_2_ between ECMO flow changes to calculate changes in blood flow (12). In this current study, we applied the same technique to continuous direct measurements of gas exchange rather than applying the technique for the differences between weaning steps. However, our results show that a steady state is essentially not reached with changing total V̇co_2_ (Supplemental Material). This results in changing conditions (e.g., total V̇co_2_ removal), which directly influences our blood flow calculations. Giosa et al. (33) have demonstrated that carbon dioxide stores are substantial and a steady state of carbon dioxide exchange may be achieved only over hours or even days. We chose 3-min measurement intervals empirically. This approximates the time resolution of a fast-responding cardiac output thermodilution system (34), but also may reduce the error introduced by a breath-by-breath system (35). Whether this measurement period could be further optimized needs to be studied further. Mainly these technical limitations of our approach may be overcome in future practice: Measurements of gas exchange at the ECMO using the Haldane transformation are accurate. Precise real-time measurements of V̇o_2_ through new technologies such as molecular inflow gas spectroscopy may substantially improve our approach and enable precision demonstrated by measurements of V̇o_2Blood_ (36). A steady state for V̇o_2_ is much more easily reached, as body stores are insubstantial, and is indirectly shown by our blood content calculations.

This data set is to our knowledge the largest documentation of gas exchange and its distribution between native and artificial lung in an experimental model of V-A ECMO. Our data show that there is a clear distribution of oxygen uptake and carbon dioxide removal between the two circuits with a respective transfer of gas exchange load through flow changes. The presented method may aid in evaluating the function of the native cardiopulmonary unit not only through monitoring of the gas exchange distribution but also through direct assessment of sufficient gas exchange transfer. The venous inlet of the ECMO circuit and the blood that flows through the pulmonary artery show substantial differences in oxygen content and carbon dioxide tension. The amount of oxygen uptake through the ECMO circuit is limited by the venous saturation, a fact that is also important for veno-venous (V-V) ECMO configurations (37). The differences between pulmonary artery and venous ECMO saturations also imply that central venous saturations are an insufficient surrogate for mixed venous saturations (Fig. 4*A*), a finding that has been reproduced by other studies (38).

In conclusion, our modified Fick principle produces clinically accurate and precise measurements of native cardiac output on V-A ECMO, when based on blood oxygen content. When other gases are used, steady state behavior and technical measurement inaccuracies may significantly contribute to imprecision. Normalizing toward a V̇/Q̇ ratio of 1 improves accuracy and precision. The inflow gas content between membrane lung and natural lung are significantly different and should be taken into account for mass balance equations on V-A and presumably V-V ECMO.

Our modified Fick principle for the estimation of cardiac output on VA-ECMO may offer a continuous measurement possibility for monitoring these patients in the future, when precision may be improved by further developments in expired gas measurement techniques. For current practice and management, and also for the development of mathematical models of ECMO gas exchange, it must be recognized that differential hypoxia is not a limited phenomenon on the arterial side, but also exists between the ECMO inflow and the pulmonary artery.

## DATA AVAILABILITY

 The data that support the findings of this study are available from the corresponding author upon reasonable request.

## SUPPLEMENTAL DATA

10.6084/m9.figshare.19070132 Supplemental Figures per Animal: https://doi.org/10.6084/m9.figshare.19070132.

10.6084/m9.figshare.20489439 Supplemental Tables S1–S4 and Supplemental Figs. S1–S3: https://doi.org/10.6084/m9.figshare.20489439.

## GRANTS

The study was supported by grants from the Stiftung für Forschung in Anästhesiologie und Intensivmedizin (No. 26/2018) awarded to K. F. Bachmann and D. C. Berger and from the Fondation Johanna Dürmüller-Bol (No. 481) awarded to K. F. Bachmann.

## DISCLOSURES 

The Department of Intensive Care Medicine of the Inselspital has, or has had in the past, research contracts with Abionic SA, AVA AG, CSEM SA, Cube Dx GmbH, Cyto Sorbents Europe GmbH, Edwards Lifesciences LLC, GE Healthcare, ImaCor Inc., MedImmune LLC, Orion Corporation, and Phagenesis Ltd. and research and development/consulting contracts with Edwards Lifesciences LLC, Nestec SA, and Wyss Zurich. The money was paid into a departmental fund; no author received any personal financial gain. Authors K. F. Bachmann, D. C. Berger, and L. Gattinoni filed a patent (PCT/EP2020/060428) for the described method, which was not prolonged. The Department of Intensive Care Medicine received unrestricted educational grants from the following organizations for organizing a quarterly postgraduate educational symposium, the Berner Forum for Intensive Care (until 2015): Abbott AG, Anandic Medical Systems, Astellas, AstraZeneca, Bard Medica SA, Baxter, B | Braun, CSL Behring, Covidien, Fresenius Kabi, GSK, Lilly, Maquet, MSD, Novartis, Nycomed, Orion Pharma, Pfizer, and Pierre Fabre Pharma AG (formerly known as RobaPharm). The Department of Intensive Care Medicine has received unrestricted educational grants from the following organizations for organizing biannual postgraduate courses in the fields of critical care ultrasound, management of extracorporeal membrane oxygenation, and mechanical ventilation: Abbott AG, Anandic Medical Systems, Bard Medica SA., Bracco, Dräger Schweiz AG, Edwards Lifesciences AG, Fresenius Kabi (Schweiz) AG, Getinge Group Maquet AG, Hamilton Medical AG, Pierre Fabre Pharma AG (formerly known as RobaPharm), PanGas AG Healthcare, Pfizer AG, Orion Pharma, and Teleflex Medical GmbH. 

## AUTHOR CONTRIBUTIONS

D.C.B., L.Z., D.C., P.P.H., M.H., L.G., and K.F.B. conceived and designed research; D.C.B., L.Z., K.N., D.C., P.P.H., H.J., and K.F.B. performed experiments; D.B., L.Z., D.C., and K.F.B. analyzed data; D.C.B. and K.F.B. interpreted results of experiments; L.Z. and K.F.B. prepared figures; D.C.B. and K.F.B. drafted manuscript; D.C.B., L.Z., K.N., D.C., P.P.H., H.J., M.H., L.G., and K.F.B. edited and revised manuscript; D.C.B., L.Z., K.N., D.C., P.P.H., H.J., M.H., L.G., and K.F.B. approved final version of manuscript. 
